# Molecular Phenogroups in Heart Failure: Large-Scale Proteomics in a Population-Based Cohort

**DOI:** 10.1161/CIRCGEN.124.004953

**Published:** 2025-07-16

**Authors:** Carolina G. Downie, Joseph J. Shearer, Kayode O. Kuku, Suzette J. Bielinski, Jorge R. Kizer, Bruce M. Psaty, Jungnam Joo, Véronique L. Roger

**Affiliations:** 1 Heart Disease Phenomics Laboratory, Epidemiology and Community Health Branch (C.G.D., J.J.S., K.O.K., V.L.R.), National Heart, Lung, and Blood Institute, National Institutes of Health, Bethesda, MD.; 2 Office of Biostatistics Research (J.J.), National Heart, Lung, and Blood Institute, National Institutes of Health, Bethesda, MD.; 3 Division of Epidemiology, Department of Quantitative Health Sciences, Mayo Clinic College of Medicine and Science, Rochester, MN (S.J.B.).; 4 Division of Cardiology, San Francisco Veterans Affairs Health Care System, and Departments of Medicine, Epidemiology and Biostatistics, University of California, San Francisco (J.R.K.).; 5 Cardiovascular Health Research Unit, Departments of Medicine, Epidemiology and Health Systems and Population Health, University of Washington, Seattle (B.M.P.).

**Keywords:** cluster analysis, heart failure, proteomics

## Abstract

**BACKGROUND:**

Heart failure (HF) is a heterogeneous syndrome with high mortality. The need for a new taxonomy of HF is recognized; up to now, such phenomapping efforts have primarily used clinical data. Proteomics offers potential for more precise phenotypic identification and mechanistic insights. However, few phenomapping studies have used this approach, and all have focused on targeted cardiovascular proteomics panels and a restricted HF ejection fraction group.

**METHODS:**

We measured over 7000 plasma proteins in a population-based cohort of 1351 patients with HF, used k-means clustering to identify distinct phenogroups, and compared their clinical characteristics and all-cause mortality.

**RESULTS:**

Three proteomics-defined phenogroups were identified, with substantial differences in survival (phenogroup 1 5-year survival probability, 65% [95% CI, 61%–68%]; phenogroup 2, 45% [40%–51%]; phenogroup 3, 26% [22%–30%]), independent of clinical characteristics. Phenogroups also exhibited differences in several measures suggesting poorer health, including NT-proBNP (N-terminal pro-B-type natriuretic peptide), kidney function, and Meta-Analysis Global Group in Chronic Heart Failure scores, but did not differ by ejection fraction or New York Heart Association class.

**CONCLUSIONS:**

Our study demonstrates that molecular phenomapping can stratify patients with HF into distinct subgroups that go beyond predefined clinical classifications.

Heart failure (HF) is a complex, heterogeneous syndrome with high morbidity and mortality.^1^ While left ventricular ejection fraction (EF) has traditionally served as the cornerstone of HF classification,^2^ issues including reproducibility in EF measurements^3^ and overlap in some clinical traits across EF categories^4^ have raised concerns about relying solely on EF for classification.^5^ This underscores the need for a mechanistic taxonomy that can improve understanding and management of HF.

In recent years, phenomapping^6,7^ studies have used machine learning to analyze clinical characteristics and define phenogroups.^8^ While these studies offer important insights, they have limitations. Most phenogroups were defined using clinical data,^8^ limiting interpretation due to the availability and biases of documented variables. Furthermore, many studies focused on a single EF category (primarily HF with preserved EF), failing to capture the full HF spectrum.^8^ Finally, while some common phenogroups emerged,^8^ they remain heterogeneous and overlapping, limiting clinical applicability.

The advent of molecular phenotyping via high-throughput assays presents an opportunity to improve understanding of HF mechanisms. However, only a few studies have evaluated proteomics-defined phenogroups, all of which used targeted disease-related panels and a single EF group.^9–12^ Our objective was to address this knowledge gap using a comprehensive assay targeting over 7000 circulating plasma proteins to identify molecular phenogroups in a population-based cohort of HF spanning the EF spectrum, and to evaluate their associations with clinical characteristics and mortality.

## Methods

Relevant phenotypic and proteomic data will be deposited in the National Heart, Lung, and Blood Institute’s BioData Catalyst upon publication. This study was approved by the Institutional Review Boards of the Mayo Clinic and Olmsted Medical Center, and informed consent was obtained from all participants. A detailed description of the Methods is provided in the Supplemental Material. A diagram of our statistical analysis approach is provided in Figure S1.

## Results

Data from 1351 patients were available for analysis, after excluding 1 patient with insufficient plasma volume and 37 patients whose samples failed SomaLogic quality control criteria. After quality control, 7151 Slow Off-Rate Modified Aptamers (SOMAmers) were available. The median SOMAmer intraassay coefficient of variation was 8.8% (interquartile range [IQR], 6.7–12.9%).^13^ After selecting the top 30% of SOMAmers with the highest median absolute deviation and filtering sets of correlated SOMAmers, 831 SOMAmers were selected for input into consensus clustering. Consensus clustering yielded an optimal *k* of 3 phenogroups, containing n=606 (phenogroup 1), n=331 (phenogroup 2), and n=414 (phenogroup 3) patients, respectively (Figure S2). Mean expression of the 831 SOMAmers, stratified by phenogroup, is provided in Table S1.

### Clinical Characteristics

Baseline clinical characteristics of the 1351 patients overall and by phenogroup are presented in Table 1. Overall, the median age was 78 years (IQR, 68–84), 31% had an EF <40%, and 48% were women, and the median follow-up time, estimated via the reverse Kaplan-Meier method^14^ was 13.9 years (95% CI, 13.3–14.2 years). Median age was slightly higher among those in phenogroup 3 (81, IQR, 75–87) compared with phenogroups 1 (75, IQR, 64–83) and 2 (77, IQR, 68–84). Several variables, including NT-proBNP (N-terminal pro-B-type natriuretic peptide), estimated glomerular filtration rate (eGFR), and Meta-Analysis Global Group in Chronic Heart Failure (MAGGIC) scores, worsened across phenogroup 1 to phenogroup 3. Atrial fibrillation prevalence was also highest in phenogroup 3 (46%), but comparable for phenogroups 1 (32%) and 2 (30%). The distributions of several comorbidities, including body mass index, diabetes, and hypertension, were similar across at least 2 of the phenogroups. In addition, the distribution of patients with EF <40% and categorized as New York Heart Association Class 3 or 4 upon enrollment was similar across phenogroups.

**Table 1.**
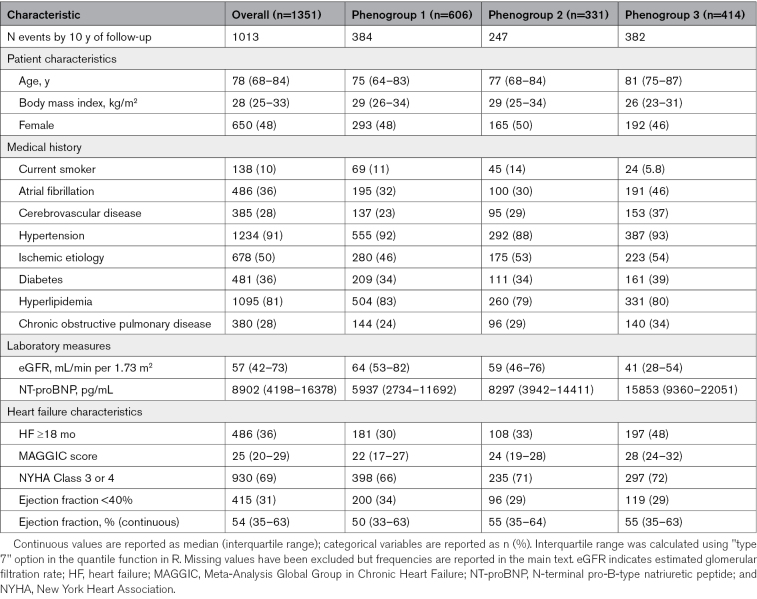
Cohort Characteristics Overall, and by Phenogroup

### All-Cause Mortality

To assess clinical relevance, we evaluated associations between phenogroups and all-cause mortality. Overall, the 5-year probability of survival (95% CI) was 48% (45%–51%). As shown in Figure A, 5-year survival probabilities varied across phenogroups, with phenogroup 1 exhibiting the highest 5-year survival probability at 65% (61%–68%), phenogroup 2 exhibiting an intermediate 5-year survival probability at 45% (40%–51%), and phenogroup 3 exhibiting the lowest 5-year survival probability at 26% (22%–30%). In contrast, 5-year survival probabilities stratified by EF were similar (EF ≥40%: 47% [44%–50%]; EF <40%: 50% [45%–55%]; Figure B). Furthermore, Kaplan-Meier survival curves of phenogroups stratified by EF showed 5-year survival probabilities within phenogroup were similar across both strata of EF (for EF ≥40%: phenogroup 1: 64% [59%–69%]; phenogroup 2: 44% [38%–51%]; phenogroup 3: 26% [21%–31%]; and for EF <40%: phenogroup 1: 66% [59%–73%]; phenogroup 2: 48% [39%–59%]; phenogroup 3: 26% [19%–35%]; Figure C and D).

**Figure.**
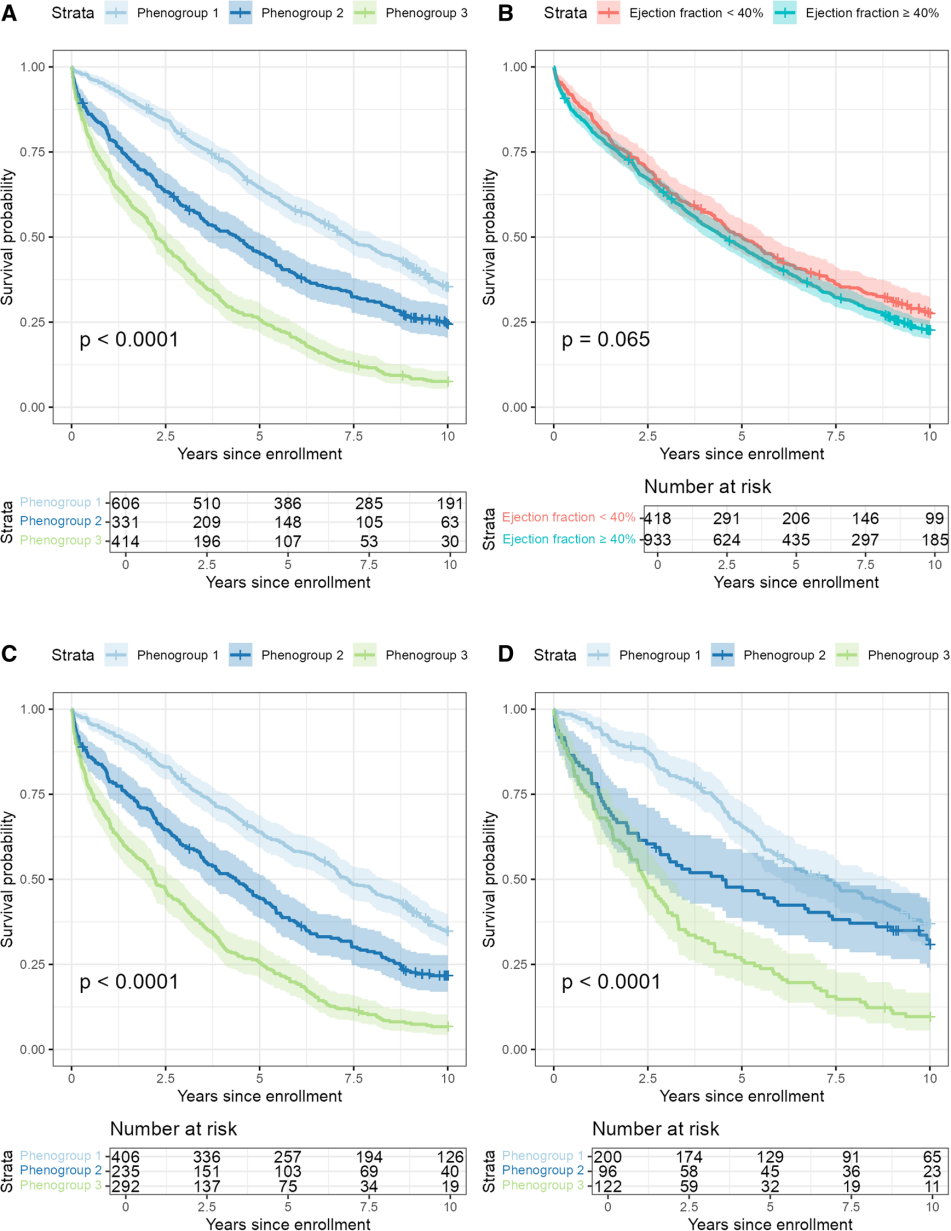
**Kaplan-Meier survival curves for all-cause mortality stratified by phenogroup assignment, ejection fraction category, and phenogroup assignment across ejection fraction category.** (**A**) Survival curves by phenogroup assignment as defined in consensus clustering for k=3. (**B**) Survival curves by ejection fraction ≥40% and < 40%. (**C**) Survival curves by phenogroup assignment among those with ejection fraction ≥40% (n=933). (**D**) Survival curves by phenogroup assignment among those with ejection fraction <40% (n=418). Number of participants at risk at each time point are displayed in tables below the Kaplan-Meier curves.

After adjustment for age, sex, and eGFR, compared with phenogroup 1, patients in phenogroups 2 and 3 had increased risk of death (phenogroup 2 hazard ratio [HR], 1.52 [95% CI, 1.30–1.79], HR_phenogroup 3_, 2.21 [1.88–2.60]; Table 2). Adjusting for NT-proBNP and MAGGIC score attenuated these estimates, but results remained statistically significant (HR_phenogroup 2_, 1.45 [1.24–1.71], HR_phenogroup 3_, 1.65 [1.41–1.94]; Table 2). Additionally adjusting for EF category yielded similar estimates (HR_phenogroup 2_, 1.41 [1.20–1.66], HR_phenogroup_ 3, 1.57 [1.33–1.84]), and interaction with EF category was not statistically significant (phenogroup 2-EF *P*-interaction: 0.94, phenogroup 3-EF *P*-interaction: 0.59, overall χ^2^ *P* for interaction=0.81). In secondary analyses, in models also adjusting for MAGGIC and NT-proBNP, adjusting for atrial fibrillation or HF duration ≥18 months yielded similar HRs, and interactions with atrial fibrillation or HF duration ≥18 months in models were also not statistically significant (phenogroup 2-atrial fibrillation *P*-interaction=0.13, phenogroup 3-atrial fibrillation *P*-interaction=0.49, overall *P* for interaction=0.32; phenogroup 2-HF duration *P*-interaction=0.39, phenogroup 3-HF duration *P*-interaction=0.30, overall *P* for interaction=0.53).

**Table 2.**
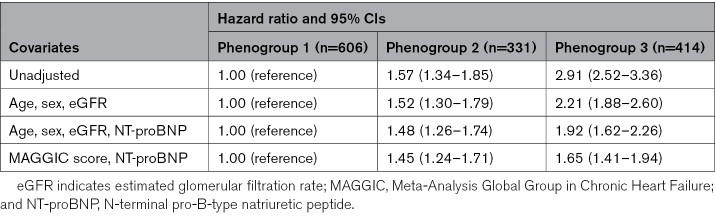
Associations Between Proteomics-Defined Phenogroup and All-Cause Mortality

### Sensitivity Analyses

In our sensitivity analysis evaluating the random selection of hub SOMAmers in sets of 3 or more correlated SOMAmers, across 1000 repetitions of selection and k-means clustering, the median concordance with our primary results was 0.981 (IQR, 0.978–0.984) and the median Rand index was 0.973 (IQR, 0.967–0.977). In sensitivity analyses evaluating 12 different combinations of median absolute deviation and correlation thresholds for dimension reduction of SOMAmers before k-means clustering for *k*=3, concordance and Rand index values comparing the resulting cluster assignments to the phenogroups defined in our primary analysis were high (concordance range: 0.87–0.96, Rand index range: 0.84–0.94). In addition, concordance and Rand index values evaluating phenogroups defined after applying multiple rounds of dimension reduction to our primary analysis SOMAmer data set were also high (concordance range: 0.91–0.95, Rand index range: 0.89–0.93). Kaplan-Meier survival curves for the 3 phenogroups generated from these sensitivity analyses were also consistent with our primary analysis (Figure S3). Furthermore, a sensitivity analysis excluding the SOMAmer targeting natriuretic peptides B (BNP) from our input set of 831 SOMAmers yielded nearly identical *k*=3 phenogroup assignments as our primary analysis (Rand index and concordance=0.999, Table S2).

### Internal Validation

Consensus clustering of the training set (n=676) yielded *k*=3 phenogroups that exhibited differences in survival comparable to our primary results, and the estimated in-group proportion was >0.72 for all phenogroups (Figure S4). After assigning phenogroups in the validation set based on training set centroids, the estimated in-group proportion was >0.73 for all phenogroups. Similar survival differences were also observed. Comparing phenogroup assignment as defined in the primary analysis to the phenogroups in the training and validation sets also yielded high concordance.

### Top Permutation Variable Importance-Ranked SOMAmers

The proteins corresponding to the top 10 permutation variable importance-ranked SOMAmers are shown in Table 3. Using these 10 SOMAmers alone as input for clustering yielded a Rand index of 0.81 and similar survival curves and HRs compared with our original phenogroups (Figure S5A and S5B). Expression of these SOMAmers varied across phenogroups, with phenogroup 3 exhibiting the highest expression levels for 6 of the top 10 SOMAmers (RANB3 [Ran-binding protein 3], SAP3 [ganglioside GM2 activator; also known as GM2A], CD59 [CD59 glycoprotein], EFNB2:ECD [ephrin-B2: extracellular domain], MIC-1 [also known as GDF-15], and SUMO2 [small ubiquitin-related modifier 2]; Figure S5C). Most of these SOMAmers had small to moderate negative correlations with eGFR (range: −0.64 [SAP3] to −0.02 [DYL2, Dynein light chain 2, cytoplasmic]), while 2 had small positive correlations (0.002 [NAA10, N-alpha-acetyltransferase 10] and 0.10 [NAP2L, nucleosome assembly protein 1-like 4]). NAA10, NAP2L, U

BQL4 [ubiquilin-4], and DYL2 all had small to modest correlations with the SOMAmer targeting BNP that was also included in the clustering analysis (range: −0.24 [NAP2L] to −0.05 [UBQL4]), while the remaining had moderate positive correlations with BNP (range: 0.35 [RANBP3 and SUMO2] to 0.46 [SAP3]).

**Table 3.**
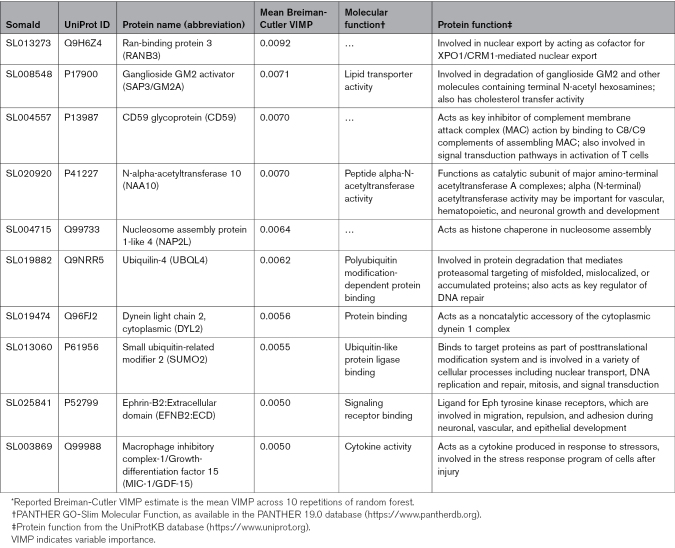
Proteins Corresponding to the Top 10 SOMAmers With Highest Variable Importance Rankings for Phenogroup Definition

All of these SOMAmers represented larger modules of correlated SOMAmers (ranging from 4-99 SOMAmers) filtered during dimension reduction. Kyoto Encyclopedia of Genes and Genomes and gene ontology pathway analysis of these modules identified several diverse pathways, including the Kyoto Encyclopedia of Genes and Genomes pathway "leukocyte transendothelial migration" for the module of 99 SOMAmers correlated with NAA10 (Table S3).

## Discussion

We identified 3 molecular phenogroups of HF using unsupervised clustering of proteomics data. These phenogroups exhibited differences in several measures suggesting poorer health status, including NT-proBNP, eGFR, and MAGGIC scores. However, New York Heart Association class, EF, and several comorbidities were similar across phenogroups. After adjustment for MAGGIC score and NT-proBNP, the phenogroups exhibited differences in survival, with membership in phenogroups 2 and 3 associated with greater mortality compared with phenogroup 1. These results add robust data to the small body of literature^9–12^ demonstrating that HF phenogroups can be identified using proteomics data. As we show here, molecular phenomapping captures underlying differences that are not reflected in measured clinical variables and are related to differences in survival. This is important because HF mortality remains high,^1^ and therefore identifying pathophysiologically relevant phenotypes and biomarkers that reflect HF risk not captured by conventional biomarkers is necessary to improve therapeutic approaches.^15^

Previous phenomapping studies differ in study populations (eg, registry-based, cohort, or trial), EF, sample size, number and types of variables included for clustering, and the number of phenogroups identified (between 2 and 15).^8^ To date, only 4 of these studies utilized proteomics for molecular phenomapping, all of which were conducted in European-based cohorts (sample size range 250–1802 participants) of a single EF type, and used targeted proteomics panels (range 92–415 protein targets).^9–12^ Although the number of identified molecular phenogroups varied (2–6), in all studies, survival differed across at least some of the identified phenogroups.^9–12^ Notably, these survival differences were not always explained by clinical variables; for example, 2 of the phenogroups reported by Woolley et al^10^ shared similar age, comorbidity, and echocardiographic patterns, yet exhibited different survival. These results are consistent with our findings: for example, median body mass index and prevalence of diabetes, hypertension, and hyperlipidemia were similar across at least 2 of our phenogroups, despite differences in survival. This demonstrates that molecular phenomapping can identify underlying differences between patients that may not be detected by assessing only clinical variables.

In their recent systematic review of over 30 published HF phenomapping studies, Meijs et al^8^ identified 9 phenogroups that repeatedly emerged. These phenogroups were summarized as: young-low comorbidity burden, metabolic, atrial fibrillation, elderly female atrial fibrillation, hypertensive-comorbidity, ischemic-male, valvular disease, devices, and cardio-renal.^8^ When comparing our 3 molecular phenogroups to these previously defined phenogroups, phenogroup 3 aligned most closely with the cardio-renal phenogroup, which was characterized in other studies by a higher frequency of older adults with chronic kidney disease and atrial fibrillation, higher NT-proBNP, and the worst survival.^8^ In our study, phenogroup 3 patients were slightly older, had lower eGFR, higher prevalence of atrial fibrillation, higher NT-proBNP values, and the worst survival. However, due to similarities in clinical characteristics as discussed above, phenogroups 1 and 2 did not as readily map onto any of the other previously commonly defined phenogroups, suggesting that molecular phenomapping captures differences in patients not reflected by clinical data.

Most previous clinical phenomapping studies were conducted in HF with preserved EF populations,^8^ and therefore could not explore whether phenogroups differed by EF. However, the few studies that included all EF categories reported minimal differences in EF by identified phenogroups,^16,17^ or minimal differences in survival by EF.^16^ Our molecular phenomapping results are consistent with these observations, as EF did not differ greatly by phenogroup, and survival across phenogroups was similar by EF strata. This suggests that the proteomic differences separating these phenogroups reflect mechanistic factors beyond EF. As calls to move beyond the paradigm of EF as the cornerstone of HF phenotyping grow,^5^ identifying and delineating characteristics, including molecular features, that differentiate patients is crucial.

The top 10 SOMAmers with the highest variable importance for defining the 3 phenogroups exhibited different expression across the 3 phenogroups and represent proteins with diverse functions, including inflammatory signaling (CD59), cytokine-involved stress response (MIC-1/GDF-15 [growth-differentiation factor 15]), and protein binding (UBQL4, DYL2). These SOMAmers did not include any natriuretic peptides and exhibited small to modest correlations with BNP; excluding the BNP-targeting SOMAmer from analysis did not change the composition of the phenogroups. This suggests that other pathways may be important in stratification of patients with HF.^15^ Though several proteins were moderately correlated with eGFR, phenogroup assignment remained significantly associated with mortality even after eGFR adjustment.

RANB3, SUMO2, MIC-1/GDF-15, CD59, SAP3, NAP2L, and EFNB2:ECD were previously individually associated with adverse outcomes in an independent HFrEF population, though only RANB3 was significantly associated with mortality in Mendelian randomization analyses, as reported by Dib et al.^18^ MIC-1/GDF-15 has been associated with cardiovascular risk factors, morbidity and mortality, and all-cause mortality among younger^19^ and older^20,21^ cardiovascular disease-free adults and individuals with HF across the EF spectrum,^22–24^ and was identified as a top protein in a previous molecular phenomapping study.^12^ However, GDF-15 and CD59 are the only proteins of our top 10 also present on at least 1 of the targeted Olink panels used in previous molecular phenomapping studies, limiting cross-study comparisons. NAA10 also represents a module of 99 SOMAmers significantly enriched for the Kyoto Encyclopedia of Genes and Genomes pathway leukocyte transendothelial migration, an important component of inflammatory processes,^25^ suggesting that inflammatory pathways could contribute to phenogroup differences. More work is needed to understand the pathophysiology underlying these phenogroups, and therapeutically targeting inflammatory pathways in HF is an active area of research^15^ that may inform eventual precision medicine approaches to HF care.

Our study has several strengths. First, we utilized an aptamer-based assay covering over 7000 proteins targets, allowing phenogroups to be based on a more comprehensive coverage of the proteome^26^ than previous proteomics-based phenomapping studies. Second, we performed this study in a community cohort larger than most previous molecular phenomapping studies^9–12^ and spanning the HF spectrum. The population-based nature of this cohort, including in- and outpatient health record data and extensive follow-up with mortality ascertainment, allowed for detailed evaluation of comorbidities and characteristics across phenogroups that are of optimal clinical relevance. Third, we found high concordance across testing and validation sets. Fourth, we conducted sensitivity analyses for dimension reduction of SOMAmers, and although we recognize that the range of thresholds we assessed does not cover the entire parameter space, they reflect a range of appropriate median absolute deviation and correlation values, and resulted in high concordance, suggesting our phenogroups were generally robust.

Some limitations should be acknowledged. It would be ideal to validate these phenogroups in an external study population. However, to our knowledge, an appropriate HF comparable to our community cohort in terms of study design and availability of SomaScan proteomics data does not exist, a limitation that is observed in other studies.^27^ However, given our internal validation results and our goal, which is focused on phenogroup discovery rather than risk prediction, we think this study constitutes an important proof-of-concept for the value of identifying HF phenogroups using large-scale proteomics. Second, we acknowledge that the dimension reduction process for selecting input SOMAmers, while necessary for achieving good performance of the clustering algorithm, limits the ability to evaluate potential causes of top-ranked SOMAmers, since highly correlated sets of SOMAmers that may reflect a variety of important biological processes were represented by a single SOMAmer. Thus, we consider our results on top permutation variable importance-ranked proteins primarily hypothesis-generating. Finally, most participants in our study identified as White, reflecting the population of the geographic area from which they were drawn.

Using the largest-to-date assay of proteomic data implemented in unsupervised clustering analysis, we identified 3 molecular HF phenogroups with differences in survival, NT-proBNP, eGFR, and MAGGIC scores. Top proteins important for defining the 3 phenogroups reflected molecular functions including stress response and inflammatory signaling. This illustrates that molecular characteristics contribute to the heterogeneity of the HF syndrome and can be used to define phenogroups, important for informing future treatment approaches.

## ARTICLE INFORMATION

### Acknowledgments

The authors extend their thanks to Dr Mary Walter and Yuhai Dai of the National Institutes of Diabetes and Digestive and Kidney Disease Clinical Laboratory Core for their assistance measuring key laboratory variables.

### Sources of Funding

C.G.D., J.J.S., K.O.K., J.J., and V.L.R. were supported by the Intramural Research program of the National Heart Lung and Blood Institute of the National Institutes of Health (ZIAHL006279). B.M.P. was supported by R01HL105756. This study also used in part the resources from the Rochester Epidemiology Project (REP) medical records-linkage system, which is supported by the National Institute on Aging (NIA; AG 058738), by the Mayo Clinic Research Committee, and by fees paid annually by REP users. The funding institution did not play a role design, conduct, analysis, or reporting nor in the decision to submit this article for publication. This research was supported in part by the Intramural Research Program of the National Institutes of Health (NIH). The contributions of the NIH authors were made as part of their official duties as NIH federal employees, are in compliance with agency policy requirements, and are considered Works of the United States Government. However, the findings and conclusions presented in this paper are those of the authors and do not necessarily reflect the views of the NIH or the U.S. Department of Health and Human Services.

### Disclosures

Dr Kizer reports stock ownership in Abbott, AbbVie, Bristol Myers Squibb, Johnson & Johnson, Lilly, Medtronic, Merck, and Pfizer. The other authors report no conflicts.

### Supplemental Material

Supplemental Methods

Tables S1–S3

Figures S1–S5

References 28–64

## Supplementary Material
